# Supplementary material for: Modelling Skylarks (Alauda arvensis) to Predict Impacts of Changes in Land Management and Policy: Development and Testing of an Agent-Based Model
Source: PLoS One. 2013 Jun 6;8(6):e65803. doi: 10.1371/journal.pone.0065803 (PMC3675089; doi:10.1371/journal.pone.0065803)
Supplement: Supporting Information S4 — The skylark ODdox as a zipped archive. (ZIP) [file pone.0065803.s004.zip › Skylark_ODdox/class_user_defined_farm26.html]

ALMaSS Skylark ODdox: UserDefinedFarm26 Class Reference


ALMaSS Skylark ODdox
 2.0


- Main Page
- Related Pages
- Classes
- Files

- Class List
- Class Index
- Class Hierarchy
- Class Members

Public Member Functions

UserDefinedFarm26 Class Reference

A farm that can have its rotation defined by the user at runtime.
More...

`#include <farm.h>`

List of all members.

|  |  |
| --- | --- |
| Public Member Functions | |
|  | UserDefinedFarm26 (const char \*fname) |
| Public Member Functions inherited from UserDefinedFarm | |
|  | UserDefinedFarm (const char \*fname) |
| Public Member Functions inherited from Farm | |
| void | AddField (LE \*a\_newfield) |
|  | Adds a field to a farm. |
| void | AddNewEvent (TTypesOfVegetation a\_event, long a\_date, LE \*a\_field, int a\_todo, long a\_num, bool a\_lock, int a\_start, bool a\_first\_year, TTypesOfVegetation a\_crop) |
|  | Adds an event to the event queue for a farm. |
| virtual bool | AutumnHarrow (LE \*a\_field, double a\_user, int a\_days) |
|  | Carry out a harrow event in the autumn on a\_field. |
| virtual bool | AutumnPlough (LE \*a\_field, double a\_user, int a\_days) |
|  | Carry out a ploughing event in the autumn on a\_field. |
| virtual bool | AutumnRoll (LE \*a\_field, double a\_user, int a\_days) |
|  | Carry out a roll event in the autumn on a\_field. |
| virtual bool | AutumnSow (LE \*a\_field, double a\_user, int a\_days) |
|  | Carry out a sowing event in the autumn on a\_field. |
| virtual bool | BurnStrawStubble (LE \*a\_field, double a\_user, int a\_days) |
|  | Burn stubble on a\_field. |
| virtual bool | CattleIsOut (LE \*a\_field, double a\_user, int a\_days, int a\_max) |
|  | Generate a 'cattle\_out' event for every day the cattle are on a\_field. |
| virtual bool | CattleIsOutLow (LE \*a\_field, double a\_user, int a\_days, int a\_max) |
|  | Generate a 'cattle\_out\_low' event for every day the cattle are on a\_field. |
| virtual bool | CattleOut (LE \*a\_field, double a\_user, int a\_days) |
|  | Start a grazing event on a\_field today. |
| virtual bool | CattleOutLowGrazing (LE \*a\_field, double a\_user, int a\_days) |
|  | Start a extensive grazing event on a\_field today. |
| virtual bool | CutToHay (LE \*a\_field, double a\_user, int a\_days) |
|  | Carry out hay cutting on a\_field. |
| virtual bool | CutToSilage (LE \*a\_field, double a\_user, int a\_days) |
|  | Cut vegetation for silage on a\_field. |
| virtual bool | CutWeeds (LE \*a\_field, double a\_user, int a\_days) |
|  | Carry out weed topping on a\_field. |
| virtual bool | DeepPlough (LE \*a\_field, double a\_user, int a\_days) |
|  | Carry out a deep ploughing event on a\_field. |
| bool | DoIt (int a\_probability) |
|  | Return chance out of 100. |
| virtual bool | FA\_AmmoniumSulphate (LE \*a\_field, double a\_user, int a\_days) |
|  | Apply ammonium supahte to a\_field owned by an stock farmer. |
| virtual bool | FA\_GreenManure (LE \*a\_field, double a\_user, int a\_days) |
|  | Spread green manure on a\_field owned by an stock farmer. |
| virtual bool | FA\_Manure (LE \*a\_field, double a\_user, int a\_days) |
|  | Spread manure on a\_field owned by an stock farmer. |
| virtual bool | FA\_NPK (LE \*a\_field, double a\_user, int a\_days) |
|  | Apply NPK fertilizer to a\_field owned by an stock farmer. |
| virtual bool | FA\_PK (LE \*a\_field, double a\_user, int a\_days) |
|  | Apply PK fertilizer to a\_field owned by an stock farmer. |
| virtual bool | FA\_Sludge (LE \*a\_field, double a\_user, int a\_days) |
|  | Spread sewege sludge on a\_field owned by an stock farmer. |
| virtual bool | FA\_Slurry (LE \*a\_field, double a\_user, int a\_days) |
|  | Spready slurry on a\_field owned by an stock farmer. |
|  | Farm (void) |
|  | Farm constructor - creates an instance of each possible crop type. |
| virtual bool | FP\_GreenManure (LE \*a\_field, double a\_user, int a\_days) |
|  | Spread green manure on a\_field owned by an arable farmer. |
| virtual bool | FP\_LiquidNH3 (LE \*a\_field, double a\_user, int a\_days) |
|  | Apply liquid ammonia fertilizer to a\_field owned by an arable farmer. |
| virtual bool | FP\_ManganeseSulphate (LE \*a\_field, double a\_user, int a\_days) |
|  | Apply Manganse Sulphate to a\_field owned by an arable farmer. |
| virtual bool | FP\_Manure (LE \*a\_field, double a\_user, int a\_days) |
|  | Spread manure on a\_field owned by an arable farmer. |
| virtual bool | FP\_NPK (LE \*a\_field, double a\_user, int a\_days) |
|  | Apply NPK fertilizer, on a\_field owned by an arable farmer. |
| virtual bool | FP\_NPKS (LE \*a\_field, double a\_user, int a\_days) |
|  | Apply NPKS fertilizer, on a\_field owned by an arable farmer. |
| virtual bool | FP\_PK (LE \*a\_field, double a\_user, int a\_days) |
|  | Apply PK fertilizer, on a\_field owned by an arable farmer. |
| virtual bool | FP\_Sludge (LE \*a\_field, double a\_user, int a\_days) |
|  | Spread sewege on a\_field owned by an arable farmer. |
| virtual bool | FP\_Slurry (LE \*a\_field, double a\_user, int a\_days) |
|  | Apply slurry to a\_field owned by an arable farmer. |
| virtual bool | FungicideTreat (LE \*a\_field, double a\_user, int a\_days) |
|  | Apply fungicide to a\_field. |
| int | GetArea (void) |
|  | Returns the area of arable fields owned by that farm. |
| int | GetFarmNumber (void) |
| int | GetIntensity (void) |
| TTypesOfFarm | GetType (void) |
| virtual bool | Glyphosate (LE \*a\_field, double a\_user, int a\_days) |
| virtual bool | GrowthRegulator (LE \*a\_field, double a\_user, int a\_days) |
|  | Apply growth regulator to a\_field. |
| virtual bool | Harvest (LE \*a\_field, double a\_user, int a\_days) |
|  | Carry out a harvest on a\_field. |
| virtual bool | HayBailing (LE \*a\_field, double a\_user, int a\_days) |
|  | Carry out hay bailing on a\_field. |
| virtual bool | HayTurning (LE \*a\_field, double a\_user, int a\_days) |
|  | Carry out hay turning on a\_field. |
| virtual bool | HerbicideTreat (LE \*a\_field, double a\_user, int a\_days) |
|  | Apply herbicide to a\_field. |
| virtual bool | HillingUp (LE \*a\_field, double a\_user, int a\_days) |
|  | Do hilling up on a\_field, probably of potatoes. |
| virtual bool | InsecticideTreat (LE \*a\_field, double a\_user, int a\_days) |
|  | Apply insecticide to a\_field. |
| bool | IsStockFarmer (void) |
| virtual void | MakeStockFarmer (void) |
| virtual void | Management (void) |
|  | Starts the main management loop for the farm and performs some error checking. |
| virtual bool | Molluscicide (LE \*a\_field, double a\_user, int a\_days) |
|  | Apply molluscidie to a\_field. |
| virtual bool | PigsAreOut (LE \*a\_field, double a\_user, int a\_days) |
|  | Start a pig grazing event on a\_field today or soon. |
| virtual bool | PigsAreOutForced (LE \*a\_field, double a\_user, int a\_days) |
|  | Start a pig grazing event on a\_field today - no exceptions. |
| virtual bool | PigsOut (LE \*a\_field, double a\_user, int a\_days) |
|  | Generate a 'pigs\_out' event for every day the cattle are on a\_field. |
| virtual bool | ProductApplication0 (LE \*a\_field, double a\_user, int a\_days) |
|  | Apply test pesticide to a\_field. |
| virtual bool | ProductApplication1 (LE \*a\_field, double a\_user, int a\_days) |
|  | Apply test pesticide to a\_field. |
| void | RemoveField (LE \*a\_field) |
|  | Removes a field to a farm. |
| virtual bool | RowCultivation (LE \*a\_field, double a\_user, int a\_days) |
|  | Carry out a harrowing between crop rows on a\_field. |
| void | SetFarmNumber (int a\_farm\_num) |
| virtual bool | SleepAllDay (LE \*a\_field, double a\_user, int a\_days) |
|  | Nothing to to today on a\_field. |
| virtual bool | SpringHarrow (LE \*a\_field, double a\_user, int a\_days) |
|  | Carry out a harrow event in the spring on a\_field. |
| virtual bool | SpringPlough (LE \*a\_field, double a\_user, int a\_days) |
|  | Carry out a ploughing event in the spring on a\_field. |
| virtual bool | SpringRoll (LE \*a\_field, double a\_user, int a\_days) |
|  | Carry out a roll event in the spring on a\_field. |
| virtual bool | SpringSow (LE \*a\_field, double a\_user, int a\_days) |
|  | Carry out a sowing event in the spring on a\_field. |
| virtual bool | StrawChopping (LE \*a\_field, double a\_user, int a\_days) |
|  | Carry out straw chopping on a\_field. |
| virtual bool | Strigling (LE \*a\_field, double a\_user, int a\_days) |
|  | Carry out a mechanical weeding on a\_field. |
| virtual bool | StriglingSow (LE \*a\_field, double a\_user, int a\_days) |
|  | Carry out a mechanical weeding followed by sowing on a\_field. |
| virtual bool | StubbleHarrowing (LE \*a\_field, double a\_user, int a\_days) |
|  | Carry out stubble harrowing on a\_field. |
| virtual bool | Swathing (LE \*a\_field, double a\_user, int a\_days) |
|  | Cut the crop on a\_field and leave it lying (probably rape) |
| virtual bool | SynInsecticideTreat (LE \*a\_field, double a\_user, int a\_days) |
|  | Apply special insecticide to a\_field. |
| TTypesOfVegetation | TranslateCropCodes (std::string &str) |
| virtual bool | Trial\_Control (LE \*a\_field, double a\_user, int a\_days) |
|  | Special pesticide trial functionality. |
| virtual bool | Trial\_PesticideTreat (LE \*a\_field, double a\_user, int a\_days) |
|  | Special pesticide trial functionality. |
| virtual bool | Trial\_PesticideTreat\_GS (LE \*a\_field, double a\_user, int a\_days) |
|  | Special pesticide trial functionality. |
| virtual bool | Trial\_ToxicControl (LE \*a\_field, double a\_user, int a\_days) |
|  | Special pesticide trial functionality. |
| virtual bool | Water (LE \*a\_field, double a\_user, int a\_days) |
|  | Carry out a watering on a\_field. |
| virtual bool | WinterPlough (LE \*a\_field, double a\_user, int a\_days) |
|  | Carry out a ploughing event in the winter on a\_field. |
| virtual | ~Farm (void) |
|  | Farm destructor - deletes all crop instances and empties event queues. |

|  |  |
| --- | --- |
| Additional Inherited Members | |
| Protected Member Functions inherited from UserDefinedFarm | |
| void | AssignPermanentCrop (TTypesOfVegetation tov, int pct) |
|  | Used to assign a permanent crop to an otherwise rotational field polygon. |
| virtual void | InitiateManagement (void) |
|  | Kicks off the farm's management. |
| int | InvIntPartition (vector< tpct > \*items, int target) |
|  | Finds all possible sums of the integers in the items array. |
| Protected Member Functions inherited from Farm | |
| void | CheckRotationManagementLoop (FarmEvent \*ev) |
| virtual int | GetFirstCropIndex (TTypesOfLandscapeElement a\_type) |
|  | Gets the first crop for the farm. |
| int | GetFirstDate (TTypesOfVegetation a\_tov) |
|  | Gets the start date for a crop type. |
| virtual int | GetNextCropIndex (int a\_rot\_index) |
|  | Returns the next crop in the rotation. |
| int | GetNextCropStartDate (LE \*a\_field, TTypesOfVegetation &a\_curr\_veg) |
|  | Returns the start date of the next crop in the rotation. |
| void | HandleEvents (void) |
|  | If there are events to carry out do this, and perhaps start a new crop. |
| bool | LeSwitch (FarmEvent \*ev) |
|  | Call do function for any crop with an outstanding event. Signal if the crop has terminated. |
| void | ReadRotation (std::string fname) |
|  | Reads a rotation file into the rotation. |

---

## Detailed Description

A farm that can have its rotation defined by the user at runtime.

---

## Constructor & Destructor Documentation

|  |  |  |  |  |  |
| --- | --- | --- | --- | --- | --- |
| UserDefinedFarm26::UserDefinedFarm26 | ( | const char \* | *fname* | ) |  |

Constructor, needs rotation but also requires a parameter for farm intensity.

References Farm::m\_farmtype, and tof\_UserDefinedFarm18.

: UserDefinedFarm(fname) // 40

{

m\_farmtype = tof\_UserDefinedFarm18;

}

---

The documentation for this class was generated from the following files:

- farm.h
- farm.cpp


- UserDefinedFarm26
- Generated on Thu Jan 10 2013 13:15:38 for ALMaSS Skylark ODdox by
   1.8.1.1
